# A price and use comparison of generic versus originator cardiovascular medicines: a hospital study in Chongqing, China

**DOI:** 10.1186/1472-6963-13-390

**Published:** 2013-10-05

**Authors:** Wenjie Zeng

**Affiliations:** 1 School of Management, Chongqing Jiaotong University, No.66 Xuefu Road, Nan’an District, Chongqing 400074, China

**Keywords:** China, Generic medicine, Price comparison, Pharmaceutical expenditure

## Abstract

**Background:**

Developed countries use generic competition to contain pharmaceutical expenditure. China, as a developing and transitional country, has not yet deemed an increase in the use of generic products as important; otherwise, much effort has been made to decrease the drug prices. This paper aims to explore dynamically the price and use comparison of generic and originator drugs in China, and estimate the potential savings of patients from switching originator drugs to generics.

**Methods:**

A typical hospital in Chongqing, China, was selected to examine the price and use comparisons of 12 cardiovascular drugs from 2006 to 2011.

**Results:**

The market share of the 12 generic medicines studied in this paper was 34.37% for volume and 31.33% for value in the second half of 2011. The price ratio of generic to originator drugs was between 0.34 and 0.98, and the volume price index of originators to generics was 1.63. The potential savings of patients from switching originator drugs to generics is 65%.

**Conclusion:**

The market share of the generics was lowering and the weighted mean price kept increasing in face of the strict price control. Under the background of hospitals both prescribing and dispensing medicines, China’s comprehensive healthcare policy makers should take measures from supply and demand sides to promote the consumption of generic medicines.

## Background

In recent years, pharmaceutical expenditure worldwide has rapidly increased and attracted significant attention. The OECD published a recent report showing that pharmaceutical expenditure has grown by more than 50% in real terms during the last decade [1]. This increase is driven by a number of factors including stricter clinical targets, rising patient expectations, aging populations, and the continued launch of new premium-priced drugs [2]. Owing to their low price and comparable quality, generic medicines are used to enhance drug prescription efficiency and thus decrease expenditure [2-4]. In general, generic medicines tend to be 20%–80% cheaper than originator medicines, especially now with a greater number imported from India (where lower production and labor costs apply) into Europe [3].

In addition, multiple supply- and demand-side reforms have been instigated in various European countries for existing drugs. Supply-side reforms include pricing reforms such as volume agreements with payback mechanisms for over budget situations and compulsory price cuts, as well as measures to obtain low prices for generics [5-11]. Demand-side measures, collated under the four Es—education, engineering, economics, and enforcement [5]—include general practitioners’ incentives to prescribe generic drugs [12-14], pharmacists’ use of generics in substitution for originator drugs [15-18], and consumers’ attitudes to generic drugs [17-20]. These measures have resulted in some progress; e.g., there has been an increase in the use of generics in Portugal [21], generic substitution has been welcomed in Finland [17], and there are incentives for pharmacists’ to dispense generics in France [22]. However, there is a continuing need for countries to look to the experiences of other nations to determine which measures are most appropriate for them [21].

China is also challenged by the increased financial pressure in medicine use, especially with the development of its health care reform. Since the initiation of economic reforms in 1979, the social and economic structures associated with the health sector reform have undergone significant changes. This has resulted in a decreased reliance on state funding, an increase in the cost of medical care, reduced public financing, and increased funding by user fees. In addition, individual health insurance cover in China decreased from approximately 70% in 1981 to only 20% percent in 1993. However, this rate increased with the 2003 implementation of a new insurance scheme, namely the New Rural Cooperative Medical Insurance Scheme. In 2009, the total medical insurance coverage rate was 93% [23]. As a proportion of total health expenditure, out-of-pocket payments rose from 20% in 1978 to 60% in 2001, and then fell to 36% in 2010. Outpatient and inpatient expenditure on pharmaceuticals from 1990 to 2010 are displayed in Table 1. In 2010, the pharmaceutical revenue of Chinese public hospitals amounted to 405.39 billion CNY (approximately US$62.37 billion), representing 45.80% of total business revenue [24].

**Table 1:** Average per capita pharmaceutical expenditures for patients in comprehensive hospitals (RMB)

**Year**	**Average medical charges for per outpatient**	**Medicine fee**	**Proportion%**	**Average medical charges for inpatient**	**Medicine fee**	**Proportion%**
1990	10.9	7.4	67.9	473.3	260.6	55.1
1995	39.9	25.6	64.2	1667.8	880.3	52.8
2000	85.8	50.3	58.6	3083.7	1421.9	46.1
2005	126.9	66	52.1	4661.5	2045.6	43.9
2008	146.5	74	50.5	5463.8	2400.4	43.9
2009	159.5	81.2	50.9	5951.8	2619.8	44
2010	173.8	88.1	50.7	6525.6	2834.4	43.4

It must also be noted that the supply chain of generic medicines in China has its own characteristics. Owing to the changing economic system, the Chinese government reformed its pharmaceutical distribution network from a centrally controlled supply system to a market-oriented system. Thus, a competitive mechanism has been introduced into the pharmaceutical market, and acts to improve the availability of pharmaceuticals. Under this supply chain, domestic pharmaceutical production grew dramatically while numerous imported drugs began to enter into the Chinese market.

### Products

Companies that wish to supply generic medicines are now asked to provide information regarding bioequiavailability before they can obtain authorization to enter the market. From 1986 to 2006, Chinese firms have independently developed only 40 categories of chemical medicines [25]. However, before 2007, the State Food and Drug Administration (SFDA) had approved 177,000 drug applications [25], meaning that most of the drug applications belonged to generics. In practice, the SFDA has adopted looser restrictions for drug registration, which gave so-called new drug licenses to minor reformulations or where only the dosage or packing of existing products were changed. In October 2007, to enhance the focus on drug safety while encouraging innovation, “Measures on the Administration of Drug Registration” were employed to improve the existing registration procedures by upgrading drug appraisal and approval standards.

### Manufacturers

China currently has more than 5,000 drug manufacturing firms. Most of these are small-scale pharmaceutical businesses with a scattered geographical layout and duplicated production processes. These firms produce mainly generic drugs with little development of originator pharmaceuticals. However, China’s fast growing economy and large population have meant that the pharmaceutical market has expanded tremendously, with an annual average growth of 16.1% in recent years [26]. The total output value rose from 137.1 billion RMB in 1998 to 667.9 billion RMB in 2007 [26]. After experiencing several quality issues, the government concentrated on the strict implementation of the Good Manufacturing Practice to assure product quality.

### Price

Drug prices are determined by three administration authorities according to elementary indications and cost information. For state-priced products, the National Development and Reform Commission (NDRC) sets maximum retail prices (price cap); for province- or municipality-priced products, the price management department determines the retail prices; and for other products the ex-factory and retail prices are determined by the manufacturers themselves. To address the issue of “unusually high” prices for common use or expensive medicines (e.g., antibiotics, cardiovascular drugs, and anticancer drugs) the NDRC implemented 28 price adjustments between 1997 and 2011 [27]. Before entering catalogs for hospital procurement, the above medicines are subject to tenders for provision in each province and municipality, and procurement prices for hospitals are then determined. For cardiovascular drugs, the price was changed four times between 2006 to 2011 in Chongqing Urban District: two changes were invitations for tenders in March 2006 and April 2011, and two were attributed an NDRC adjustment of maximum retail prices in January 2007 and March 2011.

### Market

By the end of 2007 China had 13,000 wholesale pharmaceutical enterprises, 341,000 retail pharmaceutical enterprises and chain store enterprises, and 554,000 rural drug supply outlets [26]. These sales agents use sales commissions, tourism, kickbacks, and gifts to hospital managers and/or doctors who purchase or prescribe their products. The commercial promotional activities and profits of multi-layered distribution is a substantial component of the total costs of pharmaceuticals.

Data from the Datamonitor Group offer an insight into China’s generic market. The Chinese generics market grew by 11.3% in 2010 to reach a value of US$19,645.3 million. The compound annual growth rate of the market in the period 2006–2010 was 16.4%. The Chinese generics market shrank by 0.2% in 2010 to reach a volume of 95.3% of total pharmaceutical volume. The compound annual rate of change of the market in the period 2006–2010 was -0.2%.

### Physicians

In China, hospital physicians prescribe and dispense drugs, which means that every hospital has had its own pharmacy, dispensing more than 80% of China’s total medicine consumption; the remaining 20% of medicine is distributed by community drug stores [26]. The current reimbursement system used in pharmaceutical expenditure in Chinese hospitals induces dispensing doctors (DDs) to overprescribe drugs to patients and to prescribe drugs that produce the greatest profit for themselves [28]. Furthermore, the profit markups that DDs might receive from drug prescriptions are determined by the price of the medicines. In a comparative study, Lim expressed that DDs prescribed more medicines to patients than their non-dispensing counterparts [29]. Research from other regions in Southeast Asia also reinforced the notion that physicians could profit by both prescribing and dispensing drugs, and that more originator drugs were prescribed than generic drugs [30].

The “Prescription Management Ordinance” promulgated by the Chinese government in 2007 specified that prescriptions should be written using generic names or INN (international nonproprietary names). However, this ordinance has done little to increase the use of generics. For example, prescription sheets indicate brand name or manufacturer name, or these are already selected in the hospital’s electronic information system. Although prescription sheets can be filled in community pharmacies, and generic substitution for originator prescriptions is common in developed countries, such practices are rare in Chinese hospitals and community pharmacies.

Owing to the high number of pharmaceutical factories in China and low-priced products, it is hoped that the use of generics will eventually increase. However, the generic market in China has its own characteristics. In this paper, a dynamic perspective is taken with principal cardiovascular drugs to reveal the current situation concerning generic use, market share, and price comparisons. This will provide us with the fundamental information required to explore potential measures to promote the use of generic drugs in China.

## Methods

### Data source

As stated above, under the current situation of no SPD, 80% of drugs are sold in hospitals, and drug prices for hospital procurement are determined by a tender process in each province and municipality. Price variations for hospital drugs exist in each district, even in the same city, and these prices are constantly changing. Therefore, we chose data from one restricted district, i.e., Chongqing, to compare price and use evolutions.

Chongqing is a municipality directly under China’s central government. It is located in Southwest China with a total population of 23 million people. The leading therapeutic drug classes consumed in its hospitals were identified as anti-infective agents, and medicines for cardiovascular, digestive, and nervous systems [31]. Because of frequent price fluctuations, product variations, policy changes, and specific prescribing patterns, anti-infective agents were not suitable for this research. In contrast, cardiovascular drugs involve many pharmaceuticals for chronic diseases, and their long-term usage with relatively stable products made them suitable for this study, and in the study period the drug price was the most important factors affecting the utilization structure. Other researchers have also studied this kind of medicine [7,32]. We chose a typical hospital in which to conduct our investigation, i.e., a hospital affiliated to the Third Military Medical University, which is one of the largest hospitals in Southwest China. It is also a typical health provider because of the services it provides to the public.

The dataset was obtained from the magazine company of China Pharmacy (an authoritative Chinese Journal). The magazine company is located in Chongqing, which makes it easy for it to collect detailed information from the larger hospitals in Southwest China. The dataset contains all individual delivery information including product name, purchase date, dosage form, specification, manufacturer, unit price, and purchase volume, which was collated from the hospital’s procurement records.

### Selection of generic and originator medicines

In this research, originator medicines are referred to as products once possessing intellectual property, and almost all these medicines are global products provided by international pharmaceutical enterprises including Bayer, Pfizer, and AstraZeneca et al. Generic drugs are domestic chemical products produced by enterprises with local investments, and most of the medicines are in strong competition with a number of manufacturers.

A set of different drug classes (each class containing one originator and one or more generic drugs of the same chemical entity) were chosen to compare their market share and price evolution. Based on our dataset and other reports, the three most prescribed drug groups in cardiovascular medicines were chosen for this study, including calcium channel blockers with mainly vascular effects, sartans, and statins [7,32]. Of the 19 matched products, 12 products were used in this study. Some products with relatively high usage rates were not used (such as Fluvastatin, Pitavastatin, and Losartan) because they lacked data from domestic generic products.

### Price calculation, comparison

Ordinarily, three kinds of drug prices are used in price comparison research, i.e., ex-manufacturer price, wholesale price, and retail price. The retail price policy of the hospital pharmacy has been adjusted several times, and the markup has decreased from 50% to approximately 15% (in some districts it has been reduced to zero in 2012). In this paper, the hospital’s procurement price was adopted to avoid eventual retail price discrepancies. The Chinese currency renminbi (RMB) “*yuan*” is used to determine the price and value, and the exchange rate was RMB 6.12 to US$1.00 on September 12, 2013.

We employed defined daily doses (DDDs) as a standard unit of measurement according to the World Health Organization definitions of these 12 products [33,34], and used the price per DDD (DDDc) to empirically measure the procurement price set. That is, we transformed the directly observable procurement price (*P*_p_) into DDDc using a equation: DDDc = (*P*_p_/Q) × DDD, where *Q* indicates the dosage unit per package (or strength of product).

The prices of the individual and overall 12 products were assayed by adopting a weighted mean price, which were calculated by dividing market value by market volume. For the price comparison of generic and originator drugs in 2011, the price index (*P*_*volume*_) was calculated according to the weighted volume of the procurement (ω_i_).

Pvolume=∑i=1nPoriginators'Pgenerics×ωi

$$\omega_{i}=\frac{{\mathrm{Volume}}_{i}}{\sum_{i=1}^{n}{\mathrm{Volume}}_{i}}$$

where Volume_*i*_ indicates the total volume (DDDs) of a medicine, and p_originator_ or p_generics_ indicates the weighted mean price (DDDc, *P*_*w*_).

We took six months as the analysis period, because one year is too long to express the volume change and one quarter is too short to remove possible anomalies. And we used “1H” to represent the first half year and “2H” for the second half year.

## Results

### Market share of generic and originator drugs

**Figure 1:**
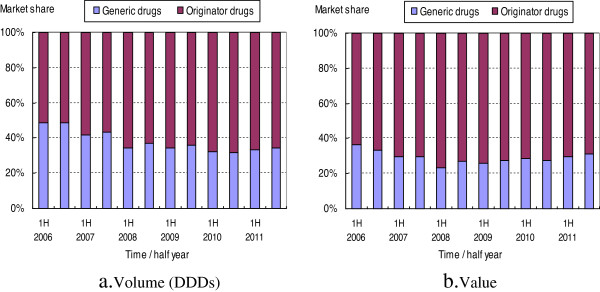
**Market shares of originator and generic cardiovascular drugs from 2006 to 2011.** The market shares were displayed with 100% stacked column chart. Each panel indicates the percentage of originator and generic drugs, and the cumulative proportion of each stacked element always totals 100%.

The market share for volume of generic drugs decreased from approximately 50% in the first half year of 2006 to 34.37% in the second half year of 2011, and that of originator drugs increased to 65.63% (Figure 1). A continuous decrease in generics market share could be observed in the early years until it reached its stable platform in the first half year of 2008 and maintained narrow fluctuations in the following years.

The evolution of the market share for value was more complicated. The value share of generic drugs also steadily decreased in the early years until it reached its valley floor (23.52%) in the first half of 2008, and then it increased slowly to 31.33%.

### Price and use evolution

**Figure 2:**
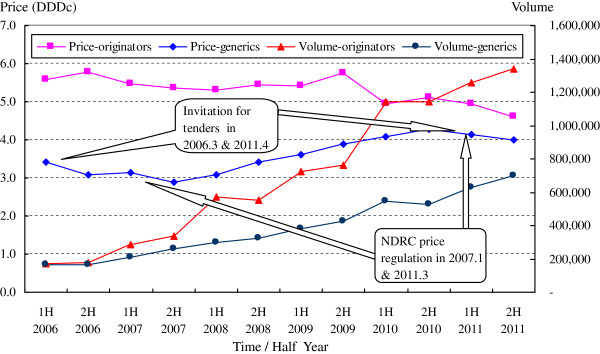
Overall weighted mean price (DDDc) and volume (DDDs) of 12 cardiovascular medicines from 2006 to 2011.

The overall weighted mean price (DDDc) and total volume (DDDs) of 12 cardiovascular medicines from 2006 to 2011 were calculated and presented in Figure 2. The half-year total volume of generic and originator drugs increased continuously, but the increase rate of originator drugs was much higher than that of the generics. The weighted mean price of generics was also in a rising channel, in contrast with the originator drugs, which attributed to the sharp price decline of Simvastatin by its manufacturer Merck Co. to compete for market share in the end of 2009.

### Price comparison and potential savings

Because some of the products were not purchased, especially generic drugs in the early study years, we employed the latest data (2011) to compare the price of generic and originator medicines.

We used the proportion of the individual volume in the total volume as the weighted index, and calculated the price comparison index. The overall prices of the 12 generic and originator drugs are displayed as DDDc in Table 2. The ratios of DDDc of generic to originator medicines ranged from 0.34 to 0.98. The volume index of originator to generics was 1.63. Thus, the price of originator drugs is 63% higher than generics.

**Table 2:** Price comparisons of 12 generic and originator drugs in 2011 (DDDc, RMB)

**Medicine**	**Prices of generics**	**Number of local manufacturers**	**Prices of originators**	**Global manufacturer**	**Generics to originators ratio**
Nifedipine	1.57	4	4.47	Bayer	0.35
Amlodipine	4.68	4	4.78	Pfizer	0.98
Felodipine	1.61	1	3.27	AstraZeneca	0.49
Lacidipine	1.27	1	3.68	GlaxosmithKline	0.34
Simvastatin	2.94	1	3.97	Merck	0.74
Pravastatin Sodium	5.80	1	7.04	Sankyo	0.82
Atorvastatin Calcium	7.73	2	9.22	Pfizer	0.84
Irbesartan	3.21	1	4.47	Sanofi-Synthelabo	0.72
Irbesartan and Hydrochlorothiazide	3.19	1	4.47	Sanofi-Synthelabo	0.71
Losartan Potassium and Hydrochlorothiazide	3.90	1	5.89	Merck	0.66
Valsartan	2.41	1	5.76	Novartis	0.42
Telmisartan	1.74	1	2.44	Boehringer Ingelheim	0.71

For all 12 brand-name drugs, generic drugs were available in the identical form with lower prices. We could estimate the potential savings from more consistent use of generic drugs for each individual medication in the following way:

$$\begin{array}{l} \mathrm{Potential}\;\mathrm{saving}s_{\mathrm{originator}}\\ \;=\;\sum \left[\mathrm{DDD}s_{\mathrm{originator}}\times \left(\mathrm{DDD}c_{\mathrm{originator}}-\mathrm{DDD}c_{\mathrm{generics}}\right)\right] \end{array}$$

The potential savings from general substitution of these 12 originator drugs in 2011 is 4.19 million RMB, with a proportion of 66% for originator utilization.

### Principal generic products

**Figure 3:**
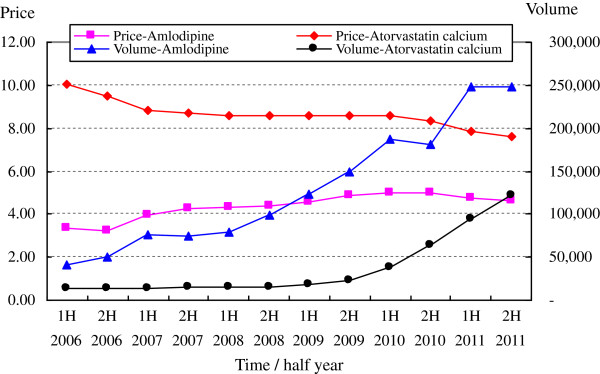
Price (DDDc) and volume (DDDs) evolution of generic amlodipine and atorvastatin calcium.

Among the generic drugs with the highest DDDc (with the exception of Pravastatin Sodium launched late in 2009), two products, i.e., Amlodipine and Atorvastatin Calcium, showed significant increase of use in the study period, especially in recent years (see Figure 3). The market shares of these two products were 43.01% and 30.80% for general value in 2011, which were much higher than for other products. This reveals an extremely abnormal phenomenon in generic drug use. Otherwise, the discrimination in the market share of originator drugs was much lower, ranging from 4.10% to 17.82% in value, with the only exception of Lacidipine at just 0.73%.

## Discussion

The results in this paper reveal the current situation regarding 12 generic cardiovascular medicines in a hospital in China:

--Total volume: There was an obvious increase in the use of both generic and originator medicines. In the second half of 2011, the volume of prescribed generics was 4.27 times that in the beginning of the study period, and 7.72 for originators.

--Market share: In the second half of 2011, the market share of the 12 generic medicines decreased to 34.37% for volume (DDDs) and 31.33% for value, compared with 48.64% (volume) and 36.65% (value) at the beginning of the study period.

--Price comparison: The price ratios of generic to originator drugs were between 0.34 and 0.98, and the volume price index of originator to generics was 1.63. Thus, the price of originator drugs is 63% higher than generics for volume. The weighted mean price of generics shows an increasing trend.

--Special products: Two principal generic products, i.e., amlodipine and atorvastatin calcium, occupied 43.01% and 30.80% of the market share in generic value, respectively. These shares are substantially higher than those enjoyed by other generic products.

As a general trend, pharmaceutical consumption in China has experienced a significant increase in recent years. The increase in cardiovascular drug consumption can be attributed to a number of reasons, including urbanization, an aging of population accompanied by an increase of chronic diseases, the expansion of the scope of medical insurance, and improvements in living standards. Using the experience of other countries for reference, generic substitution can decrease pharmaceutical expenditure, with a potential saving of 66% for patients by switching from originator to generic drugs. This result is similar to the results from a study on four generic medicines in China (which showed a 65% saving) [35], and a further study that found a 60% reduction in costs where patients used generic substitutions in 17 middle-income countries [36].

However, the increasing trend of the weighted mean price of generics, the lowering market share of generics, and the fact that just two principal generic products enjoy a substantially higher market share, can be largely explained by changes in the consumption structure, suggesting that more higher priced drugs were prescribed than cheaper ones by hospital physicians (this can be observed in Figures 1 and 2 by contrasting the evolution of the volume and value market share of generic and originator drugs). For example, the abnormal phenomenon in generic drug use of two principal generic products might be attributed to the relatively higher DDDs, which would bring greater economic interests to the whole supply chain from the prescribing and dispensing of these two drugs.

We can learn two lessons from these results. The first concerns the current state of the Chinese health sector, where hospitals prescribe and dispense drugs. From another point of view, this paper also reveals the attitudes of DDs to generic medicines, because most drugs are consumed according to doctors’ prescriptions. Owing to the financial incentives of DDs as incompetent agents and nontransparent price and quality information for patients, DDs may prescribe more expensive drugs than needed.

The second implication regards the drug price policy employed in China in recent years. Although the direct price cut or invitation for tenders can affect drug costs in the short term, the pharmaceutical supply chain adjusts the product structure and increases the weighted mean price in the long term. In contrast, the average price of medicines has dropped in recent years by 43.18% in European countries with high generic market shares and by 21.56% in low market share countries [3].

Generic competition, in general, changes the market and decreases the price of medicines. The creation of a sustainable generic pharmaceutical market requires active regulatory and marketing measures at all levels including incentives for manufactures, physicians, and dispensers [7,37]. For China’s comprehensive healthcare policy makers, there is a long way to go before an appropriate system to promote the consumption of generic medicines can be established, especially under the background of hospitals both prescribing and dispensing medicines. Potential measures should stem from supply and demand sides, including a corresponding policy for greater transparency in the pricing of generics, restrictive prescription regulations for physicians, health education to ensure the greater acceptance of generics by patients, and a reimbursement system for the prescription of generics [38,39].

This research is subject to some limitations, for example, the data collection was from just one region, one hospital, and one indication; this occurred because data from community drugstores are not easy to collect. The market share of generics may not represent all hospitals, but the price ratio of originator to generics did reveal the situation in Chongqing hospitals, and thus does show the current situation in Chinese hospitals. In addition, the effect of traditional Chinese medicine was not included in this study, which might have a substantial effect on the usage structure of chemical products. Furthermore, some new drugs were also launched during the study period from 2006 to 2011; these were few and any effect was not apparent.

## Conclusions

This study is the first dynamic exploration of the price and use comparison of generic and originator drugs in a Chinese hospital. The market share of the generics was lowering and the weighted mean price kept increasing in face of the strict price control. And two principal generic products with relatively high DDDc occupied most of the market shares in generic value, suggesting an extremely abnormal phenomenon in general utilization. Under the background of hospitals both prescribing and dispensing medicines, China’s comprehensive healthcare policy makers should take measures from supply and demand sides to promote the consumption of generic medicines.

## Competing interests

The author declare that they have no competing interests.

## Pre-publication history

The pre-publication history for this paper can be accessed here:

http://www.biomedcentral.com/1472-6963/13/390/prepub
